# Pomegranate (*Punica granatum*) Juice Shows Antioxidant Activity against Cutaneous Leishmaniasis-Induced Oxidative Stress in Female BALB/c Mice

**DOI:** 10.3390/ijerph14121592

**Published:** 2017-12-18

**Authors:** Badriah Alkathiri, Manal F. El-Khadragy, Dina M. Metwally, Ebtesam M. Al-Olayan, Muhammed A. Bakhrebah, Ahmed E. Abdel Moneim

**Affiliations:** 1 Department of Zoology, College of Science, King Saud University, Riyadh 11451, KSA; badara25.ba@gmail.com; 2 Chair Vaccines Research of Infectious Diseases, Faculty of Science, King Saud University, Riyadh 11451, KSA; eolayan@ksu.edu.sa; 3 Department of Zoology and Entomology, Faculty of Science, Helwan University, Cairo 11795, Egypt; boudy33sa@gmail.com; 4 Parasitology Department, Faculty of Veterinary Medicine, Zagazig University, Zagazig 44519, Egypt; 5 Life Science & Environment Research Institute/Director, National Center for Genome Technology, KACST, Riyadh 12371, Saudi Arabia; mbakhrbh@kacst.edu.sa

**Keywords:** pomegranate (*Punica granatum*), cutaneous leishmaniasis, lesions, treatment

## Abstract

*Leishmania* species are parasites that multiply within phagocytes and cause several clinical diseases characterized by single or multiple ulcerations. One of the complications that can induce tissue damage and the resulting scars is caused by secondary bacterial infections. Studies to find new, effective, and safe oral drugs for treating leishmaniasis are being conducted since several decades, owing to the problems associated with the use of antimonials available. Previously, the antiparasitic and antioxidant properties of *Punica granatum* (pomegranate, *P. granatum*) have been reported. Therefore, in the present study, we aimed to investigate the antileishmanial activity of pomegranate aqueous juice in vitro and in female BALB/c mice. A 3-(4.5-dimethylthiazol-2-yl)-2,5-diphenyltetrazolium bromide (MTT) assay in *Leishmania major* promastigotes and alterations in the antioxidant status, liver function, and skin histological changes in *L. major*-infected mice orally treated with pomegranate juice alone and in combination with the antibiotic ciprofloxacin, were used to investigate the in vitro and in vivo antileishmanial activity of pomegranate juice, respectively. Oral *P. granatum* juice treatment significantly reduced the average size of cutaneous leishmaniasis lesions compared with that of the untreated mice. This antileishmanial activity of *P. granatum* was associated with enhanced antioxidant enzyme activities. Histopathological evaluation proved the antileishmanial activity of *P. granatum*, but did not reveal changes in the treated animals, compared to the positive control. In conclusion, *P. granatum* shows high and fast antileishmanial activity probably by boosting the endogenous antioxidant activity.

## 1. Introduction

Leishmaniasis is a neglected disease that causes severe public health problems worldwide. It is caused by several species of the flagellated protozoa belonging to the genus Leishmania, and is transmitted by the female sand fly [1]. This disease is observed in three main forms: cutaneous, visceral, and mucocutaneous [2]. Several drugs are available for the treatment of cutaneous leishmaniasis; however, these drugs cause adverse effects. Furthermore, the rate of spontaneous healing depends on many factors, including lesion location and the presence of secondary bacterial infections [3]. Surface epithelial ulceration and localized dermal infiltrate composed of mixed acute and chronic non-specific inflammatory cellular infiltrates, containing macrophages, lymphocytes, and neutrophils, were observed in infected, untreated mice. Numerous *Leishmania* amastigotes were seen either inside or outside the macrophages, causing blood vessel congestion and interfering with the blood supply through inflammatory cellular infiltration [4].

Currently, effective vaccines against leishmaniasis that control the disease are not available [5]. Furthermore, antileishmanial chemotherapy is limited to a few compounds such as Pentostam (sodium stibogluconate). The exposure of macrophages to *Leishmania* leads to the generation of reactive oxygen species (ROS) and reactive nitrogen species (RNS), which contribute to the regulation of the inflammatory response controlled by the cellular antioxidant defense system [6]. Several internal or external pathological factors such as viral, bacterial, and parasitic infections disrupt the oxidant/antioxidant balance, leading to oxidative stress, including the oxidation of lipids, proteins, and nucleic acids [7,8]. ROS accumulation in cells can damage membrane lipids, which are probably the most susceptible cell components, if not prevented by an appropriate antioxidant scavenging system. 

The antioxidant defense system is closely associated with nutrition. Some exogenous low-molecular weight antioxidants play a crucial role and can be obtained from an appropriate diet. The exogenous and endogenous antioxidant defense systems act in coordination, with their levels being regulated by each other, to avoid oxidative stress events [9]. In the past few decades, a considerably large group of antioxidant molecules that are widespread in plants has come into focus.

Pomegranate (*Punica granatum*, *P. granatum*), belonging to the family Punicaceae, is one of the oldest known edible fruit [10]. It is extensively cultivated in the Mediterranean area and in the near and Far East countries. This botanic isolation is coincident with a unique biochemistry. Pomegranate seeds contain oil consisting of 80% punicic acid, a rare trans 18-carbon fatty acid [11]. The seeds also contain the highest botanical concentration of the sex steroid, estrone, at a concentration of 17 mg/kg dried seed [12]. Pharmacological properties of pomegranate extracts, such as antimicrobial, anti-parasitic, antiviral, and anticancer effects, have been studied previously [12]. Therefore, in the present study, we investigated the protective role of *P. granatum* aqueous juice against *Leishmania major*-induced oxidative damage in mice.

## 2. Materials and Methods 

### 2.1. L. major Isolate

Promastigotes of a Saudi sub-strain of *L. major* (ZymowmeLON4) were used and maintained in 25 mL culture flasks containing Roswell Park Memorial Institute (RPMI)-1640 medium supplemented with fetal bovine serum (FBS; Sera Laboratories International, Horsted Keynes, UK), 100 U/mL penicillin + 100 mg/mL streptomycin, and 1% l-glutamine. Each flask was incubated on its side in a standard incubator set at 25 °C. This incubation method increases medium aeration, thus allowing the cells to recover and grow faster [13].

### 2.2. Plant Material

Pomegranate (*P. granatum*) fruits were purchased from a market in Riyadh, Saudi Arabia. The plant material was authenticated in the Botany Department, on the basis of taxonomic characters and direct comparison with the specimens available at the Botany Department herbarium.

### 2.3. Pomegranate Juice Preparation

Ten kilograms of pomegranates were washed with running tap water and manually peeled, without separating the seeds. Juice was obtained using an electric blender (Braun, Frankfurt, Germany) and filtered (Whatman no. 1 filter paper). The resulting filtrate was immediately diluted with distilled water (1:1) and stored at –20 °C for no longer than two months [11].

### 2.4. Pomegranate Juice Stability

Pomegranate juice stability was assessed by measuring the initial total phenolic content of the juice and evaluating the alterations after two and three days of exposure to the same conditions as the juice supplied to the animals. The total polyphenol content was determined following the standard Folin-Ciocalteu method.

### 2.5. Anti-Promastigote Assay Using MTT (In Vitro Assay)

Different dilutions of pomegranate juice or Pentostam (GlaxoSmithKline, London, UK) were prepared in RPMI medium. Exponential-phase *L. major* promastigotes in cultured media (1.5 × 10^6^/mL) were seeded into a 96-well plate and treated with the desired dilutions of the drugs/juice (10–200 μL/mL). Promastigotes in the control wells were not exposed to any treatment. Blank wells contained only RPMI media. The experiments were performed in duplicate. Plates were incubated at 26 °C in 5% CO_2_ for 48 h and a modified MTT colorimetric assay was performed for the detection of promastigote viability. Here, 250 μg/mL MTT reagent (Sigma-Aldrich, St. Louis, MO, USA) was added to each well, plates were incubated for 4 h at 26 °C, and dimethyl sulfoxide (DMSO) was added to dissolve the formazan crystals. The amount of tetrazolium salts cleaved to formazan, which directly correlates with the number of metabolically active promastigote in the culture, was quantified using a plate reader at 540 nm absorbance [14]. Promastigote viability was calculated using the following equation:

Cell viability = (Ab_treated_/Ab_untreated_) × 100

### 2.6. Experimental Protocol

Sixty female BALB/c mice (age: 5–6 weeks; weight: 25–30 g) were obtained from the Animal House of King Saud University, Faculty of Science (Riyadh, Saudi Arabia). The animals were housed in wire-bottomed cages in a room under standard conditions of illumination with a 12 h light-dark cycle 55 ± 5% relative humidity and 25 ± 2 °C for one week until treatment initiation. They were provided with tap water and balanced diet *ad libitum*. All animals received human care in compliance with the state authorities following the Saudi Arabia rules of animal protection. The study protocol was approved (IRB Number: K.S.U-2017-722/PI) by Ethical Committee of King Saud University (Riyadh, KSA). The mice were allocated randomly to six experimental groups (n = 10 mice/group) as follows:

Group l: Normal non-infected negative control group.
Group 2: Infected un-treated positive control group: Mice were subcutaneously inoculated with 1 × 10^7^ promastigotes in a shaved area above the tail.
Group 3: Infected mice treated with Pentostam (Pen; 120 mg/kg subcutaneously) for four weeks starting with the first appearance of an ulcerative lesion.
Group 4: Infected mice treated with 0.8 µg/mL pomegranate (Pom; *P. granatum*) juice for four weeks starting with the first appearance of an ulcerative lesion.
Group 5: Infected mice treated with 0.8 µg/mL pomegranate (*P. granatum*) juice concurrently with the antibiotic ciprofloxacin (CIP, 10 mg/mL) for four weeks, starting with the first appearance of an ulcerative lesion.
Group 6: Mice pretreated with 0.8 µg/mL pomegranate (*P. granatum*) juice for four weeks before infection.

The treatment was initiated when local lesions were apparent. The mice were treated daily for four weeks. Each week, the lesion size before and after treatment was measured with Vernier caliper. Parasitemia was determined every alternate day by observing lesion appearance (3–4 weeks post infection). Mortality was checked daily. Effects on ulcerative lesions were assessed clinically. Ulcer cure was defined as clinical improvement based on reduction in lesion size compared with the lesion size of untreated infected control mice.

### 2.7. Measurement of Lesion Size 

Two diameters (L and W; at right angles to each other) of the lesions were measured using Vernier calipers, and the size (mm^2^) was determined according to the formula established previously [15]:

Lesion size (LS) = (L + W)/2

### 2.8. Liver Function Test

The amount of alanine aminotransferase (ALT) and aspartate aminotransferase (AST) in mouse sera was determined according to the method described by Reitman and Frankel [16]. 

### 2.9. Oxidative Stress Markers

Skin homogenates were prepared in 50 mM Tris-HCl, pH 7.4, and lipid peroxidation (LPO) to thiobarbituric acid reactive substances (TBARS) was assessed using the method described by Ohkawa et al. [17]. Additionally, the homogenates were used to determine the levels of nitrite/nitrate (nitric oxide; NO) [18] and glutathione (GSH) [19].

### 2.10. Enzymatic Antioxidant Activities

Superoxide dismutase (SOD) activity in serum was determined by the inhibition of its colorimetric reaction using an SOD assay kit (Cayman Chemical, Ann Arbor, MI, USA) according to the previously described methods [20] and the absorbance at 460 nm was measured with a plate reader (Spectramax 250, MTX Lab Systems, Bradenton, FL, USA). Serum catalase (CAT) activity was measured using a CAT assay kit (Cayman Chemical), as described previously [21].

### 2.11. Gene Expression Profile by RT-PCR (Real-Time Polymerase Chain Reaction) in Skin

Total RNA was extracted from the skin tissue by the TRIzol method, according to the manufacturer’s protocol, as previously described [22]. The quantity and integrity of RNA were measured using a nanodrop. The isolated RNA had an A 260/280 ratio of 1.9–2.1. Then, first-strand cDNA was synthesized from 1 μg total RNA by reverse transcription with a SuperScript^TM^ first-strand synthesis system kit (Invitrogen, Carlsbad, CA, USA), according to the manufacturer’s instructions. Real-time PCR was performed according to the method described by Alshabanah et al. [22]. The PCR primer sequences were BLAST (Basic Local Alignment Search Tool)-searched to ensure specificity to the desired gene and are provided in Table 1. We used β-actin as the endogenous control. 

### 2.12. Histological Changes

The skin was fixed in 10% neutral buffered formalin for 24 h, dehydrated in ethyl alcohol, cleared in xylene, and mounted in molten paraplast. Sections with 4–5 µm thickness were stained with hematoxylin-eosin and examined using a Nikon microscope (Eclipse E200-LED, Tokyo, Japan).

### 2.13. Statistical Analysis

Differences between obtained values (mean ± standard error of mean (SEM)) were assessed by one-way analysis of variance (ANOVA) followed by the Duncan’s multiple range test. Differences with *p* values of 0.05 or less were considered statistically significant. 

## 3. Results

### 3.1. In Vivo Cytotoxicity of Pomegranate Juice 

**Figure 1:**
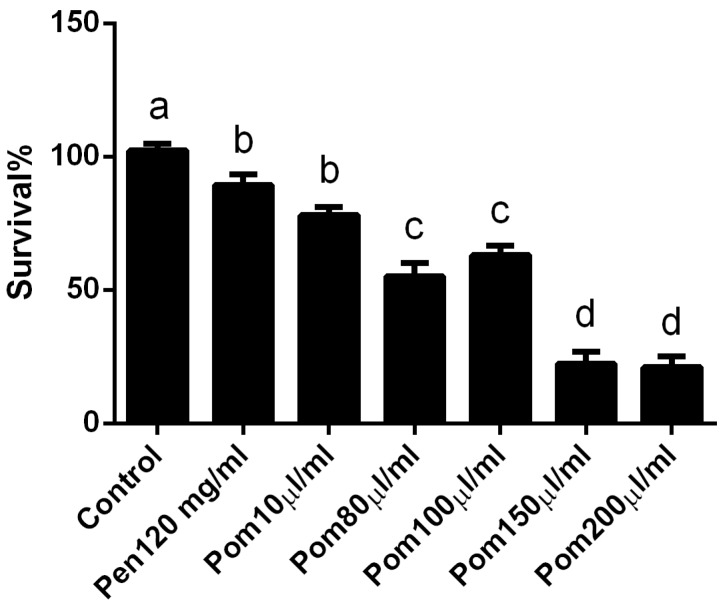
Viability of *Leishmania major* promastigotes in the presence of various concentrations of the pomegranate juice (Pom) and Pentostam (Pen) as a positive control after 48 h incubation. Means indicated with different letters differ significantly.

The in vitro cytotoxic potential of pomegranate juice against *L. major* promastigotes was tested using the MTT assay to determine 50% inhibitory concentration (IC_50_). As illustrated in Figure 1, pomegranate juice showed a dose-dependent cytotoxic effect with almost 83.7% death at a concentration of 200 µL/mL in the current study, we found that the percentage of growth inhibition to be increased with increasing the concentration of pomegranate, and IC_50_ value of the juice was 118.2 µg/mL.

### 3.2. Antileishmanial Activity of Pomegranate Juice

**Figure 2:**
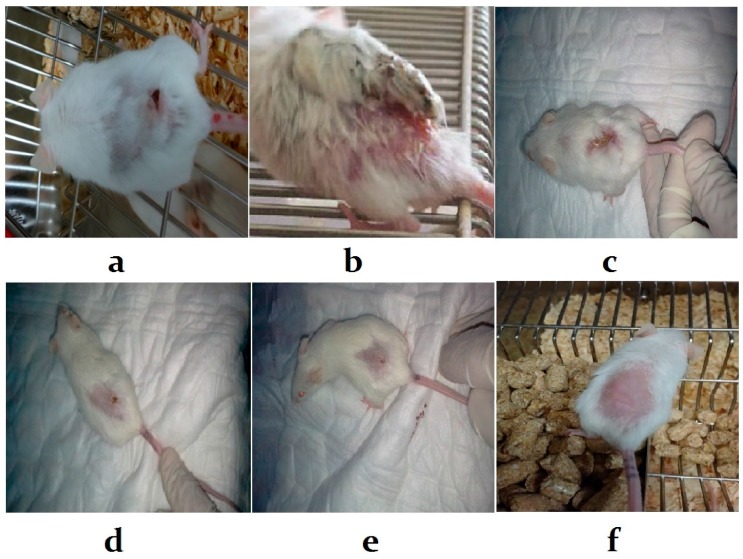
Cutaneous lesions of infected mice in with or without of pomegranate juice supplementation. (**a**) Three weeks of post-infection untreated mouse showing redness, swelling and ulcer formation. (**b**) Seven weeks post-infection untreated mouse showing redness, swelling, ulcer and gangrene formation. (**c**) Three weeks post-infection treated with Pentostam showing redness, swelling, ulcer and gangrene formation, however, the formed lesion was smaller than that of untreated mice. (**d**) Three weeks post-infection treated with pomegranate showing restored to normal skin with small ulcer. (**e**) Three weeks post-infection treated with pomegranate and CIP showing restored to normal skin with small ulcer. (**f**) Four weeks post-infection pretreated with pomegranate showing restored to normal skin.

**Figure 3:**
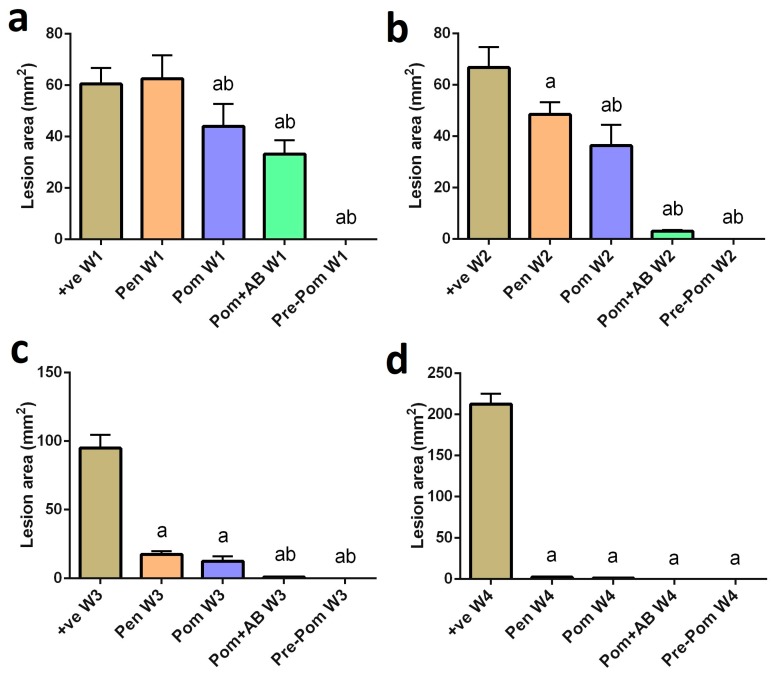
Lesion size in mouse skin one (**a**), two (**b**), three (**c**), and four (**d**) weeks after infection. The mice were treated with saline (+ve), Pentostam (Pen), pomegranate juice supplementation alone (Pom) or pomegranate juice with antibiotic (Pom + AB) or pre-treated with pomegranate juice (Pre-Pom). Lesion sizes were measured with a digital caliper as described in the Material and Methods. ^a^ *p* < 0.05, significant change compared to +ve Control group; ^b^ *p* < 0.05, significant change compared to Pentostam group.

In all the infected female BLAB/c mice, cutaneous lesions developed and increased in size. Gangrene started to develop at four weeks post-infection (Figure 2a,b), whereas, autoamputation was observed in infected animals treated with pomegranate juice group as well as those treated with pomegranate and CIP (Figure 2d,e). These results indicate that pomegranate juice pretreatment had remarkable dose-dependent antileishmanial activity against *L. major* promastigote (Figure 2f). Moreover, the antileishmanial activity of pomegranate is more potent than that of Pentostam (Figure 2c). The mean lesion area of infected untreated mice increased gradually to 15.2 mm^2^ at the fourth week of the experiment (Figure 3). In infected mice treated with pomegranate juice alone, the swelling gradually decreased to 1.2 mm^2^ (two weeks post-treatment) and the skin appeared normal with no clinical relapse four weeks post-treatment as compared to the infected untreated mice. Similarly, in infected mice treated with pomegranate juice and CIP, the swelling gradually decreased to 0.5 mm^2^ (two weeks post-treatment) and the skin became normal with no clinical relapse two weeks post-treatment. No skin changes were observed in mice pretreated with pomegranate juice, indicating that pomegranate juice is effective for treatment of *L. major*. Pentostam treatment was required for more than 28 days, despite which the mice did not show full recovery and only reduction in lesion size was observed. Thus, the antileishmanial activity of pomegranate juice was higher than that of the standard drug.

### 3.3. Effect on Liver Function after Pomegranate Juice Treatment 

**Figure 4:**
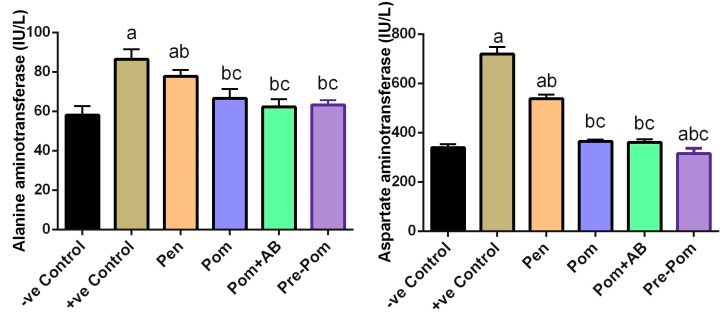
Effect of pomegranate juice supplementation and Pentostam treatment on the activities of serum transaminases in the control and experimental groups four weeks after infection. Values are mean ± SEM (n = 10). ^a^ *p* < 0.05, significant change compared to –ve Control group; ^b^ *p* < 0.05, significant change compared to +ve Control group; ^c^ *p* < 0.05, significant change compared to Pentostam group.

Liver function parameters, viz. ALT and AST levels, were significantly increased in *L. major*-inoculated mice, compared to the normal non-infected mice (*p* < 0.05; Figure 4). The levels of these markers were almost reversed to the control levels in the groups treated with pomegranate juice alone and with pomegranate juice plus CIP, as well as the pretreated group. However, Pentostam treatment failed to reverse this effect. 

### 3.4. Antioxidant Activity of Pomegranate Juice

**Figure 5:**
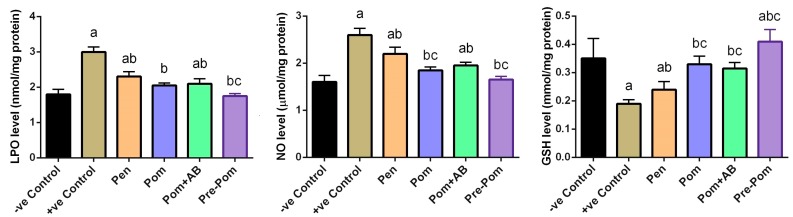
Effect of pomegranate juice supplementation and Pentostam treatment on oxidative stress markers in the control and experimental groups at four weeks after infection. Values are mean ± SEM (n = 10). ^a^ *p* < 0.05, significant change compared to the –ve Control group; ^b^ *p* < 0.05, significant change compared to +ve Control group; ^c^ *p* < 0.05, significant change compared to Pentostam group. LPO: lipid peroxidation; NO: nitric oxide and GSH: glutathione.

To examine the effect of *L. major* infection on the oxidant/antioxidant status, LPO, NO, and GSH levels in skin of mice were determined (Figure 5). Compared to the control mice, mice inoculated with *L. major* showed a significant (*p* ˂ 0.05) increase in the levels of LPO and NO with a concomitant decrease in GSH content in skin. Pentostam treatment significantly decreased the levels of these oxidant markers, compared to those in the infected mice; however, these decreased levels were significantly higher than those observed in the control mice. Interestingly, pomegranate juice treatment alone, before or concurrently with the infection, attenuated the reduction of GSH and the formation of LPO and NO in the skin (Figure 5), whereas pomegranate juice and CIP treatment partially prevented the reduction of GSH and the elevation of LPO and NO in the skin.

**Figure 6:**
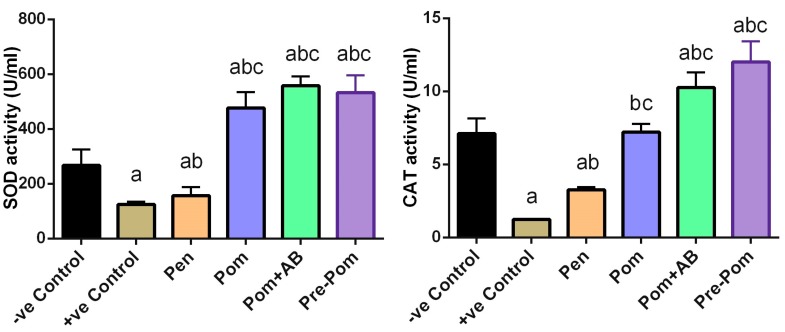
Effect of pomegranate juice supplementation and Pentostam treatment on dermal antioxidant enzyme activities in the control and experimental groups at four weeks after infection. Values are mean ± SEM (n = 10). ^a^ *p* < 0.05, significant change compared to –ve Control group; ^b^ *p* < 0.05, significant change compared to +ve Control group; ^c^ *p* < 0.05, significant change compared to Pentostam group. SOD: superoxide dismutase and CAT: catalase.

**Figure 7:**
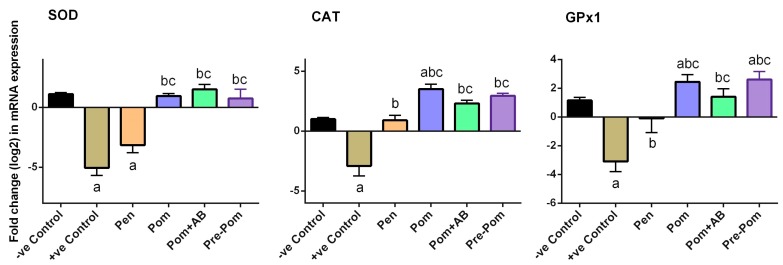
Effect of pomegranate juice supplementation and Pentostam treatment on dermal mRNA expression of candidate genes in the control and experimental groups. Results (mean ± SEM, n = 3) were normalized to β-actin RNA level and are shown as fold induction (in log2 scale) relative to the mRNA level in the control group. ^a^ *p* < 0.05, significant change compared to –ve Control group; ^b^ *p* < 0.05, significant change compared to +ve Control group; ^c^ *p* < 0.05, significant change compared to Pentostam group. SOD2: superoxide dismutase 2, CAT: catalase and GPx1: glutathione peroxidase 1.

Further, to prove the effect of leishmaniasis on the oxidant/antioxidant imbalance in the dermal tissue, the alteration of antioxidant enzymes was studied, including the activity of SOD and CAT enzymes. Compared to the control mice, mice infected with *L. major* showed a significant reduction in the activities of these antioxidant enzymes (*p* < 0.05; Figure 6). In contrast, compared to the untreated infected mice, mice treated with pomegranate juice, before or concurrently with the treatment, showed significant increase in the activities of SOD and CAT (*p* < 0.05). Furthermore, combined treatment with pomegranate juice and CIP partially prevented the reduction of these antioxidant enzyme activities. Consistent with the biochemical results, the quantitative RT-PCR results showed that mRNA expression of SOD, CAT, and GPx1 in the skin of *L. major*-infected mice were downregulated, compared to those in the control mice (Figure 7). 

### 3.5. Histological Examination

**Figure 8:**
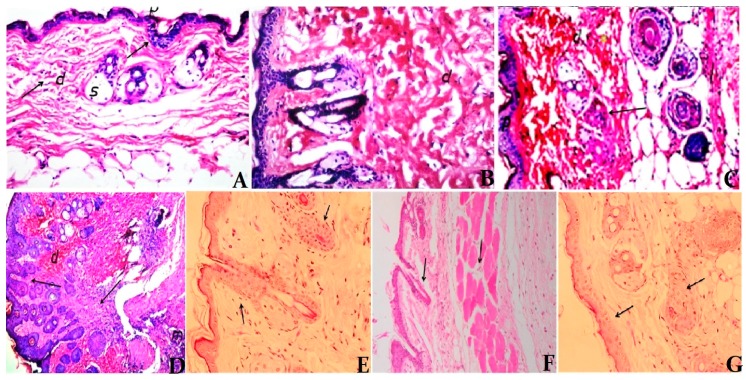
Hematoxylin and eosin-stained skin sections at four weeks post infection. (**A**) Control group, showing the normal histological structure of the epidermis, dermis, subcutaneous tissues and musculature layer (×200). (**B**) Untreated infected group, showing intact histological structure of the epidermis (P) and dermis (d), and no histopathological alteration in both epidermal and dermal layers (×400). (**C**) Untreated infected group, showing suppurative liquefactive necrosis with pus formation in deep subcutaneous tissue (×400). (**D**) Pentostam-treated group, showing acanthosis in dermal layer and infiltration of inflammatory cells in both the dermal layer (d) and subcutaneous musculature tissue (m) (×400). (**E**) Pomegranate juice-supplemented group, showing normal histological structure of epidermis and dermis and infiltration of few inflammatory cells in the dermal and subcutaneous tissue (×200). (**F**) Pomegranate juice-supplemented with antibiotic treated group showing normal histological structure of the epidermis and dermis layers (×400). (**G**) Pre-pomegranate juice-supplemented group, showing normal histological structure of the epidermis and dermis layers (×400).

Examination of the histological changes in the skin of mice from all the groups supported the results observed from other experiments (Figure 8). Four weeks after infection, the skin of infected mice showed a moderately dense, localized dermal infiltrate composed of mixed acute and chronic nonspecific inflammatory cellular infiltrates. Congested dilated blood vessels were observed, and numerous *Leishmania* promastigotes were seen either inside or outside the macrophages. Moreover, suppurative liquefactive necrosis was observed in deep subcutaneous tissue. In Pentostam-treated mice, the skin showed normal histological structure of subcutaneous musculature tissue and infiltration of few inflammatory cells in the subcutaneous tissue. However, apparent ameliorations were noticed in the pomegranate-treated groups; the tissue sections showed an intact epidermis with a moderately dense infiltrate, milder grade of infection in terms of both the inflammatory response and the number of visible amastigotes. Moreover, the intensity of infection was also less than that of the infected untreated controls. Surprisingly, no sign of pathological change was found in mice pretreated with pomegranate juice and those treated with pomegranate juice and CIP.

## 4. Discussion

The diagnosis of leishmaniasis in experimental animals depends on appearance of skin ulcers and the presence of amastigotes in clinical samples. Several studies failed to distinguish between the different *Leishmania* spp., because of their homogeneous morphologies [23]. Therefore, PCR is generally used to detect *L. major* infection, because it is a sensitive method and has been effective in the diagnosis and accurate treatment of leishmaniasis. Intralesional injection is the most effective treatment for mice; multiple experiments showed that 85% of all treated mice were cured [24]. In addition, Pentostam has been reported to be effective in curing leishmaniasis caused by *L. major* [25]. Recently several plant antioxidants have been studied extensively. Furthermore, pomegranate has been shown to support the skin’s underlying structure and lowers the synthesis of collagen-degrading enzymes, resulting in younger-looking skin [26]. In the present study, we demonstrate that pomegranate juice improves lesion healing and parasite resolution in BALB/c mice infected with *L. major*.

In all of the infected mice, cutaneous lesions began with redness and swelling at the site of inoculation, post infection. The swelling increased progressively followed by crust formation, as observed previously [4]. Subsequently, cutaneous ulcers developed and increased in size; then, gangrene started to develop by the end of fourth week post infection. The evolution of cutaneous leishmaniasis in mice was attributed to the accumulation of infected macrophages at the site of intradermal inoculation, after which granulocytes and lymphocytes appeared and ulceration occurred, as reported previously [4,27,28]. However, in the present study, no crust formation, ulceration, gangrene, or autoamputation were observed in the mice treated with pomegranate juice. The remarkable reduction in the inflammatory response was similar to that reported in a previous study [4], which showed a decline in the number of promastigotes and inflammatory reaction caused by *Leishmania*.

In the current study, pomegranate juice exhibited remarkable antileishmanial property. This activity may be attributed to the presence of phenolic and flavonoids compounds especially luteolin, ellagitannins and epigallocatechin gallate. The leishmanicidal activity of epigallocatechin gallate (EGCG) was greater than the standard antileishmanial drug, of miltefosine. Hence EGCG has been successfully used for the treatment of New World leishmaniasis [29]. Ellagitannins possesses antileishmanial potency by enhancing non-specific immunity by macrophages activation and inducing NO, interferon-gamma and tumor necrosis factor-gamma, thereby those chemicals and cytokines produce fundamental host defense system and kill the invading parasite [30]. Whereas, luteolin possesses leishmanicidal activity by inhibiting the extracellular promastigotes [31].

*L. major* causes inflammation by mast cell stimulation and by enhancing the secretion of pro-inflammatory mediators. ROS produced during an inflammatory response leads to oxidative injure in non-infected cells. During oxidative damage, some free radicals that play an important role in collagen damage are released [32,33]. Natural products have made and are continuing to make important contributions in the search for new leishmanicidal drugs [32,34]. 

LPO occurs because of oxidative stress resulting from the ROS and RNS over-production due to host defense against the parasite infection [35]. LPO has been implicated by free radicals responsible for cellular damage [36]. Moreover, the robust production of free oxygen radicals (O_2_^•−^) depletes the protective antioxidant enzymes, resulting in the cell injury that observed in *Leishmania* infection [37]. Increased LPO in erythrocytes has been described in visceral leishmaniasis in hamsters [38] and humans [39]. Heidarpour et al. [40] observed a significant elevation in serum LPO levels in the liver and kidney of dogs infected with *L. infantum*. The LPO level in patients with active cutaneous leishmaniasis was significantly higher in healthy subjects [41,42]. Prevention of LPO after pomegranate juice treatment could be attributed to the radical-scavenging effect of the antioxidant constituents of pomegranate juice [36]. 

The NO radicals play a crucial role in inducing inflammatory response, and their toxicity propagated only when they react with O_2_^•−^ radicals to form peroxynitrite, which damages biomolecules such as proteins, lipids, and nucleic acids [43]. In the present study, we showed that pomegranate juice restrained NO generation, thus suggesting its applicability as a potent and novel therapeutic agent for scavenging NO. This juice may also affect the regulation of pathological conditions caused by excessive generation of NO and its oxidation product peroxynitrite. Enhanced levels of NO and peroxynitrite have been reported in the blood and lesions respectively, of mice infected with *L. amazonensis* [44].

Cutaneous leishmaniasis leads to a significant reduction in the GSH level, which can be an important factor in the toxicity induced by the parasite. GSH is the main nonenzymatic antioxidant molecules found in cells and plays a protective role in the metabolism of several toxic agents. It acts as a free radical-trapping agent and to preserve cytochrome P450 by blocking LPO [45]. Our findings are in agreement with the results reported by Jafari et al. [7]. Infection with the Iranian strain of *L. major* (MRHO/IR/75/ER) depleted GSH in the skin and lungs of infected BALB/c mice. In the current study, *P. granatum* juice markedly increased the and maintained GSH level. The increase in GSH level is important for GPx1, which requires GSH as a cofactor; the elevation in GSH level increases GPx1 activity. The current study showed reduction in GPx1 levels. 

A significant decrease in CAT and SOD activities in cutaneous leishmaniasis has been reported [46]. The results of SOD, GPx1, and CAT expression experiments indicate that ROS production was highly elevated during cutaneous leishmaniasis, thus further confirming that free radicals and oxidative damage certainly play a vital role in the pathogenesis of injury; this also provides strong evidence for the effectiveness of natural antioxidants in the treatment of toxic injury. This pattern is similar to the previous results from studies on mice spleen tissue infected with *L. major* [7,8]. Several studies reported reduced activities of CAT and SOD in the erythrocytes of hamsters [47] and humans [48] and a significant decrease in serum total antioxidant status in the liver and kidney of dogs infected with *L. infantum* [40]. In our study, treatment with *P. granatum* juice caused a significant increase in CAT and SOD activities. This may be attributed to host defense for protection against toxic oxygen metabolites secreted by the parasite. *P. granatum* juice treatment prevented the reduction in the antioxidant enzymes, similar to the results of previous studies that have shown a positive effect of different classes of polyphenols and flavonoids on antioxidant enzyme activities in vivo [36,49].

Our findings reveal that pomegranate juice supplemented alone caused a significant increase in SOD and CAT. These results are in accordance with those reported by Faria et al. [50]. Some polyphenols are known to be able to modulate the transcription and expression of proteins involved in endogenous antioxidant defense, by interacting with antioxidant response elements in the promoter regions of protein-coding genes [51].

## 5. Conclusions

These findings indicate that oral treatment with pomegranate juice caused complete healing. The leishmanicidal activity of pomegranate juice is mainly triggered by its antiinflammatory and antioxidant activities, which benefit the infected host. Therefore, pomegranate juice is a possible new drug for cutaneous leishmaniasis and, would provide a safer, quicker, more easily administrable, and less expensive treatment for cutaneous leishmaniasis.

## Figures and Tables

**Table 1:** Primer sequences of genes analyzed by real time PCR.

Name	Accession Number	Sense (5′–3′)	Antisense (5′–3′)
β-actin	NM_031144.3	GGCATCCTGACCCTGAAGTA	GGGGTGTTGAAGGTCTCAAA
SOD2	NM_001270850.1	AGCTGCACCACAGCAAGCAC	TCCACCACCCTTAGGGCTCA
CAT	NM_012520.2	TCCGGGATCTTTTTAACGCCATTG	TCGAGCACGGTAGGGACAGTTCAC
GPx1	NM_017006.2	CGGTTTCCCGTGCAATCAGT	ACACCGGGGACCAAATGATG

Abbreviations: SOD2: Manganese-dependent superoxide dismutase (MnSOD); CAT: Catalase; GPx1: Glutathione peroxidase 1.
